# *Plasmodium malariae* contributes to high levels of malaria transmission in a forest–savannah transition area in Cameroon

**DOI:** 10.1186/s13071-022-05635-7

**Published:** 2023-01-25

**Authors:** Daniel Nguiffo-Nguete, Francis Nongley Nkemngo, Cyrille Ndo, Jean-Pierre Agbor, Stravensky T. Boussougou-Sambe, Luc Salako Djogbénou, Francine Ntoumi, Ayôla A. Adegnika, Steffen Borrmann, Charles S. Wondji

**Affiliations:** 1 Centre for Research in Infectious Diseases (CRID), Yaoundé, Cameroon; 2 Faculty of Sciences, University of Buea, Buea, Cameroon; 3 Department of Biological Sciences, Faculty of Medicine and Pharmaceutical Sciences, University of Douala, P.O. Box 24157, Douala, Cameroon; 4 Centre de Recherches Médicales de Lambaréné, Lambaréné, Gabon; 5 Institut Für Tropenmedizin, Eberhard Karls Universität, Tübingen, Germany; 6 Institut Régional de Santé Publique, Ouidah, Bénin; 7 University of Abomey-Calavi, Ouidah, Bénin; 8 Fondation Pour La Recherche Scientifique (FORS), Cotonou, Benin; 9 Fondation Congolaise Pour La Recherche Médicale, Brazzaville, Republic of the Congo; 10 German Center for Infection Research, Tübingen, Germany; 11 International Institute of Tropical Agriculture (IITA), Yaoundé, Cameroon; 12 Liverpool School of Tropical Medicine, Liverpool, UK

**Keywords:** Malaria, *P. falciparum*, *P. malariae*, *P. ovale*, Reservoir

## Abstract

**Background:**

Malaria control efforts are highly skewed towards *Plasmodium falciparum* while overlooking other *Plasmodium* species such as *P. malariae*. A better understanding of the role of *Plasmodium* species other than* P. falciparum* is needed to strengthen malaria elimination initiatives. The aim of the present study was to elucidate the contribution of *P. malariae* to malaria transmission in Cameroon.

**Methods:**

The study was conducted in the Ngatti Health District, a forest–savannah transition area in the Adamawa Region, Cameroon. A total of 497 individuals aged from 1 to 85 years were diagnosed with malaria in November 2020 using a rapid diagnostic test (RDT) and microscopy. Adult mosquitoes were collected between September 2019 and March 2020 by indoor aspiration and identified morphologically and molecularly. The infection status of *Plasmodium* spp. was also determined by quantitative PCR, and dried blood spots were collected from 156 participants with the aim to detect different *Plasmodium* species by nested PCR.

**Results:**

The overall *Plasmodium* prevalence was 50.3%, 51.8% and 64.7%, as detected by microscopy, the RDT and PCR, respectively. Based on the PCR results, *P. falciparum* was the most prevalent species (43%); followed by co-infections *P. falciparum/P. malariae* (17%), *P*. *falciparum/P*. *ovale* (1.3%), *P*. *falciparum/P*. *ovale/P*. *malariae* (1.3%); and then by *P. malariae* mono-infection (2.5%). The same trend was observed using microscopy, with 35% of participants infected with *P. falciparum*, 11% co-infected with *P. falciparum/P. malariae* and 4% infected with *P. malariae*. The prevalence and parasite density of malaria infection varied significantly with age group (*P* < 0.05), with the highest prevalence rate observed in children aged 6–10 years (*P* = 0.0001) while the density of *Plasmodium* infection increased significantly in children aged < 5 years compared to the other age groups (*P* = 10^−3^). Among the 757 *Anopheles* mosquitoes collected, 737 (97.35%) were *An. funestus* sensu stricto, 15 (1.9%) were *An. gambiae* and 5 (0.6%) were *An. hancocki*. The *Plasmodium* species recorded at the head/thorax level were *P. falciparum* and *P. malariae,* with a sporozoite infection rate of 8.4%; the highest sporozoite infection rate was recorded at Mibellon village (13.6%).

**Conclusion:**

The results of this study reveal the significant contribution of *P. malariae*, in addition to *P. falciparum*, to the high malaria transmission rate in this region. These findings highlight the need to deploy initiatives to also tackle this *Plasmodium* species to eliminate malaria in the region.

**Graphical Abstract:**

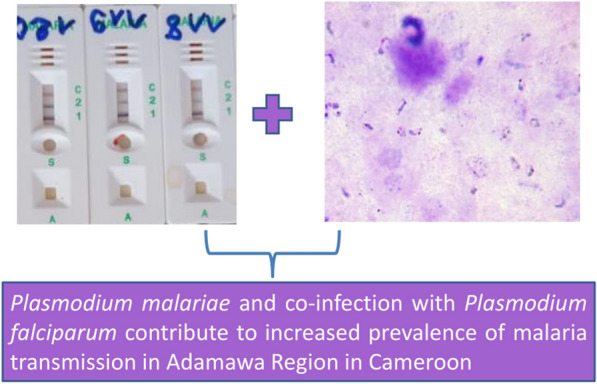

## Background

The past decades have been witness to a substantial reduction in the number of malaria cases and deaths due to malaria worldwide by 37% and 60%, respectively, between 2000 and 2015 [1]. This reduction was mainly due to the use of long-lasting insecticide-treated bed nets (LLINs), indoor residual spraying (IRS), improvements in malaria diagnosis with rapid diagnostic tests (RDTs), affordable prices and easy access to the artemisinin-based combination therapy (ACT) [1, 2].

Despite past and ongoing efforts to eliminate malaria, the disease remains endemic in some parts of the world, with > 90% of cases recorded in the sub-Saharan region of Africa. This is largely due to the increasing resistance of mosquitoes to insecticides, which has diluted the effectiveness of malaria control measures [1]. Hence, there is an urgent need to find new strategies to eliminate or even eradicate this disease. In the case of malaria, the interaction between humans and vectors contributes to maintaining the circulation of malaria in endemic areas where hosts, even if they are highly immunised, constitute the infectious reservoir for plasmodial species [3, 4]. It has been reported that 16 *Anopheles* species can be considered to vectors of malaria disease to humans in Cameroon [5–7], with the most competent vectors being *Anopheles gambiae* sensu stricto (*An. gambiae* s.s.), *An. coluzzii*, *An. arabiensis*, *An. funestus*, *An. nili* and *An. moucheti* [6, 8]. To date, the malaria control programme in Cameroon has focused on the elimination of *Plasmodium falciparum* and, to a certain degree, *Plasmodium vivax,* making them the most studied *Plasmodium* species responsible for severe cases of malaria disease. However, *Plasmodium malariae* and *Plasmodium ovale*, which have been reported to be widely distributed in most malaria-endemic regions of the world, are less studied and difficult to identify by light microscopy because the low level of parasitaemia is often below detection thresholds of microscope readers. This failure is the result of sub-microscopic malaria, which accounts for 20–50% of all human-to-mosquito transmissions in sub-Saharan Africa [9]. A *P. malariae* infection is usually benign, but could cause morbidity such as severe anaemia, acute renal failure and splenomegaly [10, 11]. It is also responsible for quartan fever and can remain in the host for decades, complicating the fight against this disease [12]. In the case of co-infection with *P. malariae and P. falciparum*, it has been reported that *P. malariae* infection can reduce the burden of parasitaemia of *P. falciparum* infection by up to 30% and contribute to the reduction of the pathology due to *P. falciparum* as the people become even more asymptomatic [13–15]. A study carried out by Yman et al. [16] in Tanzania showed a reduction of *P. malariae* prevalence when the prevalence of *P. falciparum* decreased. These results demonstrate that there is a need for more information regarding the epidemiology of non-falciparum species in the context of the distribution of malaria vaccines targeting only the *P. falciparum* antigen. Four *Plasmodium* species have been recorded to circulate in Cameroon: *P. falciparum*, *P. ovale*, *P. malariae* and *P. vivax* [5, 17–19]. *Plasmodium falciparum* remains the predominant *Plasmodium *species followed by *P. ovale and P. malariae*, with average prevalence rates of infection of 95%, 3% and 1%, respectively [5]. These prevalence rates vary according to the localities sampled and methods used to determine the species. Malaria elimination also needs to consider the identification and contribution of non-falciparum parasites to the malaria burden. Most studies to date focus on *P. falciparum* malaria and especially on symptomatic malaria cases in patients aged < 15 years [20], even though the malaria reservoir is expected to be mainly concentrated in the asymptomatic human population when the transmission is low (asymptomatic malaria was defined as the presence of *Plasmodium* in a patient with an axillary temperature of < 37.5 °C). In the study reported here, we first conducted an entomological survey to identify the vector competent to transmit *P. malariae*. Then we used molecular, microscopic and RDT techniques to evaluate the dynamics of *P. falciparum* and *P. malariae* infection transmission in all age groups of the study population.

## Methods

### Study areas

**Fig. 1:**
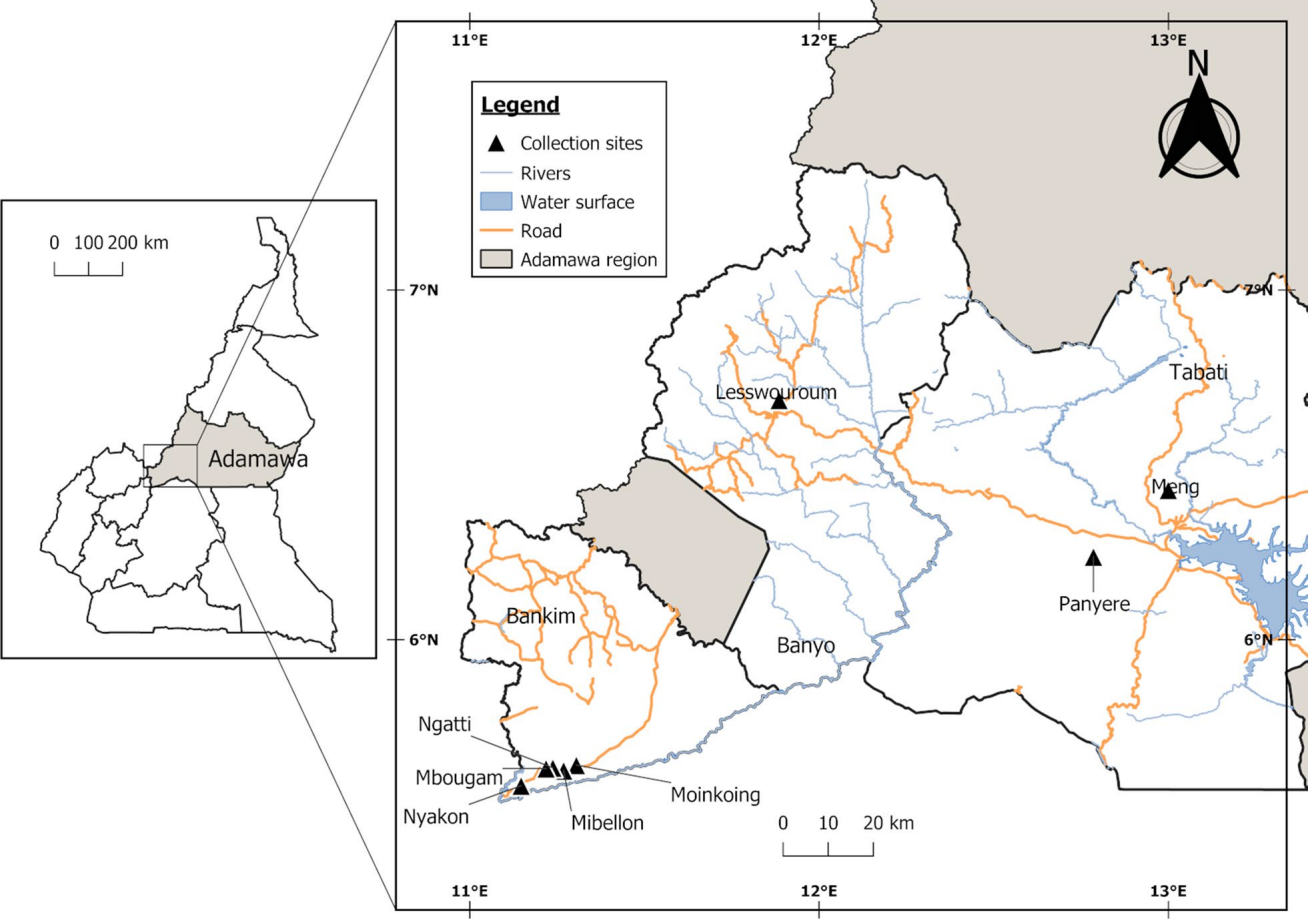
Map of collection sites

The entomological survey was performed by collecting mosquitoes in six locations between September 2019 (Nyakon, Mbougam, Mibellon, Ngatti) and March 2020 (Banyo, Panyere and Meng) (Fig. 1). The epidemiological survey was carried out in November 2020 in four villages: Moinkoing, Mibellon and Mbougam chefferie, all in the Ngatti health district located in the Adamawa Region of Cameroon, Bankim sub-division. The study carried out by Tchouakui et al. [21] showed a *P. malariae/P. ovale or P. vivax* infection rate of 4% in the mosquito population of Mibellon village, one of the villages in our study area.

### A sampling of participants

The sample size of the human population was calculated following the formula: s = z^2^ × *P* × (1 − *P*)/m^2^ [22], where s = sample size for infinite population and z = *Z*-score (a *Z*-score of 1.96 was used with a 95% confidence level), *P* = the percentage of population probability (assumed to be 50% = 0.5) and m = the margin of error (taken to be 5% = 0.05). All people from year 1 and thereafter were considered for blood collection.

### Blood collection and staining

During the survey, malaria diagnosis was carried out using thick blood smears, RDTs (CareStart™ Malaria Pf/PAN (HRP2/pLDH) Combo, Access Bio Inc. 65 Clyde Road. Somerset, NJ, 08873). and PCR analyses. Body weight, temperature, gender and age of subjects sampled in the study were taken into account. The blood was collected on slides in all the localities and on Whatman FTA cards (Sigma-Aldrich, St. Louis, MO, USA) only in Mibellon and Moinkoing villages.

Thick blood smears collected from the finger of each subject sampled in the study were air-dried and stained with 10% Giemsa solution for 20 min. The slides were screened by direct microscopy (100×) to visualise the different species of *Plasmodium* parasites. Parasite density was defined as the number of *Plasmodium* parasites per microlitre of blood, counted against 200 leukocytes and expressed according to the WHO guidelines as the number of asexual parasites per microlitre of blood, assuming a leukocyte count of 8000 cells/μl. Samples of blood drops were deposited on Whatman FTA cards and dried for 24 h at room temperature, following which they were stored in clean zippered bags until DNA extraction.

### DNA extraction

DNA was extracted from blood on Whatman paper using the Chelex protocol. Briefly, 400 µl of 5% chelex100 was added to an Eppendorf tube containing a blood sample on filter paper. The tubes were placed in a shaking dry heater and incubated at 56 °C for 1 h, following which it was incubated for 30 min at 95 °C in a shaking heater. The tube was then removed and centrifuged at a maximum of 13,500 rpm for 10 min. The supernatant was then transferred into a labelled tube and stored at − 20 °C [23].

### Real-time PCR for *Plasmodium* species identification

#### Molecular analysis

Real-time PCRs (RT-PCRs) for the identification of *Plasmodium* species infections were performed with all samples using the nested protocol with some minor modifications to differentiate the various species of *Plasmodium* [24]. A species-specific region of the 18S ribosomal DNA (rDNA) of *Plasmodium* was amplified. In the first reaction, PCR amplifications were performed in a 15-μl volume containing 1.5 μl PCR buffer A, 0.75 μl 25 mM MgCl2, 0.12 μl 25 mM dNTPs, 0.51 μl forward and reverse primers (10 mM), 0.12 μl Kapa Taq, 10.49 μl ddH2O and 1 μl DNA. A 5-μl sample of the first reaction’s product was used as a template in a second nested reaction to specifically differentiate *P. falciparum, P. vivax, P. ovale and P. malariae* products. The temperature profile for the second PCR was: 5 min at 95 °C, followed by 25 cycles of 1 min at 94 °C, 2 min at 58 °C, 2 min at 72 °C; with a final cycle at 72 °C for 10 min. The cycles were repeated 30 times for the second reaction at the same cycling parameters. After electrophoresis in 1.5% agarose gel and staining with Midori green, the amplicon products were visualized under UV light. *Plasmodium* identification was made based on amplicon sizes.

#### DNA extraction from mosquitoes, species identification and detection of *Plasmodium* parasite

Female mosquitoes were captured inside houses using an electric aspirator during the early morning hours of between 05.30 a.m. and 08.00 a.m. Female* Anopheles* mosquitoes collected were morphologically identified using morphological keys [25]. These mosquitoes were kept in a tube containing the desiccant (silica gel) for further molecular analysis. Molecular identification was performed using a cocktail PCR to determine the species [26].

All female* Anopheles* mosquitoes were dissected individually into between heads, thoraxes and abdomens. Genomic DNA (gDNA) was extracted from each body part (head + thorax and abdomen) using the LIVAK method [27], and the *Plasmodium* sporozoite infection rate was assessed using the TaqMan assay [28]. As a last step, nested PCR was conducted on all positive samples confirm the results of the Taqman assay and discriminate the *P. ovale*, *P. vivax* and *P. malariae* species [24].

### Epidemiological and statistical analyses

Data analysis was conducted using the Microsoft Excel sheet (Microsoft Corp., Redmond, WA, USA), GraphPad Prism V8.02R software (GraphPad Software Inc., San Diego, CA, USA). Data were summarised into means and standard deviations (SD), and percentages were used in the evaluation of the descriptive statistics. Proportions were compared using the Chi-square test (*χ*^2^). Parasitaemia was log transformed before analysis. Means were compared using an independent sample t-test and analysis of variance where appropriate; Kruskal–Wallis tests were used to make associations between *P. falciparum*,* P. malariae* and mixed infection and other variables of interest. *P*-values < 0.05 were considered to indicate significance, and a 95% confidence interval (CI) of geometric means that did not overlap was considered to indicate a significant difference.

## Results

### Mosquito species composition

A total of 757 *Anopheles* were collected during the study period. Three species were identified by evaluation of morphological characteristics: *Anopheles funestus* sensu stricto (*An. funestus* s.s.) (*n* = 737; 97.35%), *Anopheles gambiae* sensu lato (*An. gambiae* s.l.) (*n* = 15; 1.9%) and *Anopheles hancocki* (*n* = 5; 0.6%).

### *Plasmodium* sporozoite and oocyst infection rate

The overall infection rate for *Plasmodium* sporozoites was 8.4%, recorded in *An. funestus* s.s. and *An. gambiae* s.l. (Table 1). The highest infections rate with *P. falciparum* (10.8%),* P. OVM* (*P. malariae/P. ovale* or *P. vivax*) (2.5%) and mixed infections (*P. falciparum* and* P. OVM*) (0.4%) were recorded in Mibellong village. Mosquitoes infected with* P. OVM* were found only in the Mibellong and Tibati localities, and two mosquitoes were found to be infected with a mixed infection (*P. falciparum* and P. OVM) in Mibellong and Meng (Table 1).

**Table 1:** Prevalence of *Plasmodium* infection rate in mosquitoes

Location	Year	*Anopheles *mosquito species	Head + thorax sporozoite infection rate				
			*N*	*Plasmodium falciparum*+, *n* (%)	*P. OVM*, *n* (%)	*P. falciparum *+ *P. OVM*, *n* (%)	Total, *n* (%)
Mibellon	2019	*An. funestus* (s.s.)	279	30 (10.8)	7 (2.5)	01 (0.4)	38 (13.6)
Mbougam		*An. funestus* (s.s.)	93	5 (5.4)	0	0	5 (5.4)
Nyakon		*An. funestus* (s.s.)	93	2 (2.2)	0	0	2 (2.2)
Banyo	2020	*An. funestus* (s.s.)	20	2 (10)	0	0	2 (10)
Tibiti		*An. funestus* s.s.)	143	4 (2.7)	0	1 (0.6)	5 (3.4)
		*An. hancocki*	5	0	0	0	0
Main		*An. funestus* (s.s.)	109	9 (8.2)	1 (0.9)	0	10 (9.2)
		*An. gambiae* (s.l.)	15	2 (13)	0	0	2 (13)
TOTAL	/	/	757	54 (7.1)	8 (1)	2 (0.2)	64 (8.4)
*Abdomen (oocyst infection rate)*							
Mibellon	2019	*An. funestus* (s.s.)	279	62 (22.2)	21 (7.5)	5 (1.8)	88 (31.5)
Mbougam		*An. funestus* (s.s.)	93	13 (13.9)	5 (5.4)	0	18 (19.4)
Nyakon		*An. funestus* (s.s.)	93	8 (8.6)	04 (4.3)	01 (1.1)	13 (13.9)

* Plasmodium falciparum + : Plasmodium falciparum positive individuals, P. OVM* *P. malariae/P. ovale* or *P. vivax*,* s.l.* sensu lato,* s.s.* sensu stricto

The *Plasmodium* oocyst infection rate was considered only in samples collected in 2019, and the results showed that mosquitoes from Mibellong, Mbougam and Niakon were infected by *P. falciparum* with a prevalence of 22.2%, 13.9% and 8.6%, respectively, followed by *P.OVM* with a prevalence of 7.5%, 5.4% and 4.3%, respectively (Table 1). The mixed infection rate (*P. falciparum* and *P. OVM*) was observed only in Mibellong (1.8%) and Mbougam (1.1%).

The nested-PCR results confirmed that the samples infected by *P.OVM* were *P. malariae* for those with successful amplification (Table 1).

Taken together, these observations indicate that *P. falciparum* and *P. malariae* are circulating in these localities and, therefore, that these localities are a good place to conduct an epidemiological survey of *P. malariae.*

### Prevalence of multispecies malaria infection

For the microscopy analyses, samples were collected from 497 people (213 [42.8%] males, 284 [57.2] females) enrolled in this study. Due to some amplification issues, only 156 samples were analysed by PCR, at two locations (Mibellon and Moinkoing), from 67 (43%) males and 89 (57%) females, respectively. The prevalence of malaria infection assessed by microscopy, RDTs and PCR on the same set of samples (156) was 47.4%, 43.7% and 64.7%, respectively. The general prevalence of infection taking into account the entire study population was 51% (252/494) for microscopy and 51.8% (252/484) for microscopy (Table 2). There was a significant difference between the prevalence of *Plasmodium* spp*.* detected by PCR, microscopy and the RDT (*P* < 0.01).

**Table 2:** *Plasmodium* spp. prevalence determined by the three different methods

*Plasmodium* spp.	PCR, *n* (%)	RDT, *n* (%)	Microscopy, *n* (%)
*P. falciparum*	67 (43)	95 (19.6)	174 (35)
*P. malariae*	4 (2.5)	5 (1)	21 (4)
*P. falciparum/P. malariae*	27 (17)	151 (31)	57 (11)
*P. falciparum/P. ovale*	2 (1.3)	/	/
*P. falciparum/P. malariae/P. ovale*	2 (1.3)	/	/
Total	104 (68.4)	251 (51.8)	252 (51)

*n* number of individuals/samples, RDT rapid diagnostic test

### Prevalence of asymptomatic malaria by microscopy, RDT and PCR.

Using the same sample set used as for PCR, 93.3% (147/157) of the individuals sampled were asymptomatic. Microscopy, RDTs and PCR showed that 44.8% (95% CI: 37.0–59.1), 43.7% (95% CI: 33.9–55.6) and 62.5% (95% CI: 37.0–59.1) of asymptomatic people, respectively, were infected with *Plasmodium* spp.

### Species-specific infection prevalence

**Fig. 2:**
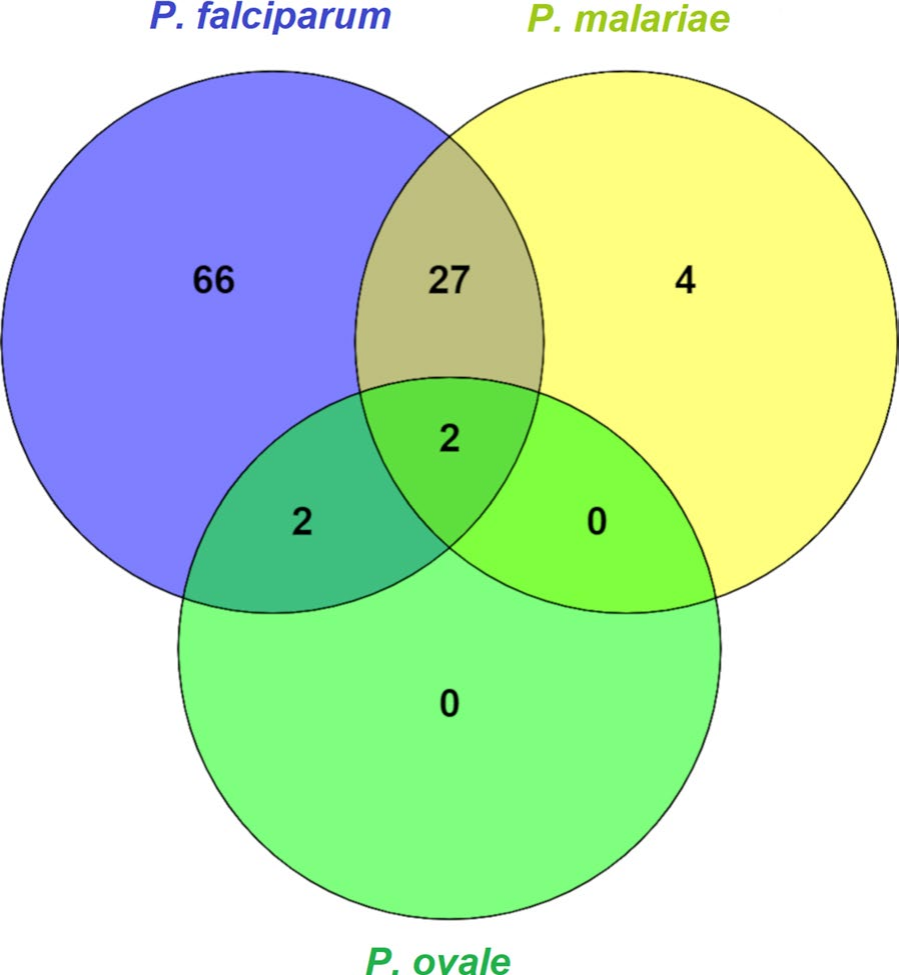
Schematic representation of the number of *Plasmodium* parasites per species (*n* = 157 samples). The circles indicate the number of samples that tested positive for each *Plasmodium* species (blue: *Plasmodium* *falciparum*; yellow: *Plasmodium* malariae; green: *Plasmodium* *ovale*). The sections where circles overlap represent the number of co-infections of each combination of > 1 *Plasmodium* species

Among the 156 samples analysed by PCR, 66 (42.3%) tested positive for *P*. *falciparum*, four (2.5%) for *P*. *malariae*, 27 (17.1%) for *P*. *falciparum/P*. *malariae,* two (1.3%) for *P*. *falciparum/P*. *ovale* and two (1.3%) for *P*. *falciparum/P*. *ovale/P*. *malariae* (Fig. 2). Also, 497 samples were analysed by microscopy, of which 174 (35%) were positive for *P*. *falciparum*, 21 (4%) for *P*. *malariae*, 57 (11%) for *P*. *falciparum/P*. *malariae* and two (0.4%) for *P*. *falciparum/P*. *ovale* (Table 3). No samples were infected with *P*. *vivax*. The general prevalence of infection detected by microscopy in Mibellon (64.8%), Moinkoing (54.6%) and Ngatti (51%) villages was high, while the lowest prevalence of 12% was recorded in Mbougam village (Table 3). Among these villages, the highest proportion of mixed infections (*P. falciparum/P. malariae*) was recorded in Mibellon (25.7%) (Table 3). Microscopy showed that *P. malariae* prevalence was 6%, 5.4%, 3.3% and 0% for Moinkoing, Mibellon, Ngatti and Mbougam chefferie villages, respectively. The prevalence of infection as assessed by PCR and per village was determined in Mibellon and Moinkoing only. The results showed an overall prevalence of 64% and 53.9%, with 3.2% and 1.5% infection with *P. malariae*, in Mibellon and Moinkoing, respectively (Table 3).

**Table 3:** Prevalence of infection according to village

Localities	*Plasmodium* infection					
		*N*	*P. falciparum*, *n* (%)	*P. malariae*, *n* (%)	*P. falciparum*/*P. malariae*, *n* (%)	Total, *n* (%)
Bougam chefferie	Microscopy	63	7 (11)	0	1(1.5)	8 (12.6)
	PCR	/	/	/	/	/
Mibellon	Microscopy	128	43 (35.5)	7 (5.4)	33 (25.7)	83 (64.8)
	PCR	93	36 (38.7)	3 (3.2)	25 (26.8)	64 (68.8)
Moinkoing	Microscopy	100	42 (42)	6 (6)	6 (6)	54 (54)
	PCR	63	31 (49)	1 (1.5)	2 (3)	34 (53.9)
Ngatti	Microscopy	206	82 (39.8)	7 (3.3)	18 (8.7)	107 (51.9)
	PCR	/	/	/	/	/

*P. falc/mal P. falciparum* and* P. malariae*

**Fig. 3:**
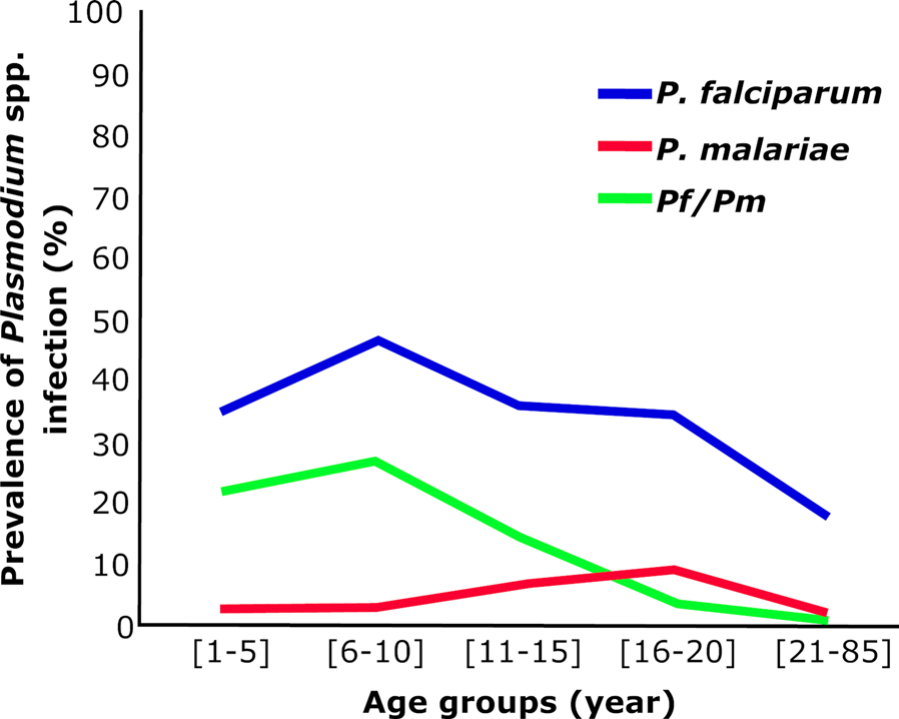
Prevalence of *Plasmodium* infection by age group.

According to age groups, the highest *P. falciparum* prevalence of 47.7% (95% CI: 34–64) was observed among older children aged 6–10 years, followed (in order of decreasing prevalence) by teenagers aged 11–15 years (36.7%, 95% CI: 44–57%), infants aged 1–5 years (34.7%, 95% CI: 26–49%), young adults aged 16–20 years (34%, 95% CI: 19–56%) and adults, with the latter age group showing the lowest prevalence of 22% (95% CI 19–26%) (Fig. 3). The prevalence of mixed infection (*P. falciparum/P. malariae)* followed the same trend, with the highest prevalence of 27% observed in children aged 5–10 years, followed (in order of decreasing prevalence) by subjects in the age groups 1–5, 10–14, 16–20 and > 21 years, with 22%, 14.2%, 4.5% and 1% infection rates, respectively (Fig. 3). For *P. malariae* mono-infection, the highest prevalence (9%) was recorded in subjects aged 16–20 years, followed a prevalence of 7.1% in subjects aged 11–15 years. The prevalence of *P. malariae* obtained in subjects aged 6–10, 1–5 and 21+ years was 3.4%, 3.3% and 2%, respectively (Fig. 3). In general, the probability of having a high prevalence of infection with *P. falciparum* and mixed infections (*P. falciparum/P. malariae*) was highest in children aged 6–10 years (odds ratio [OR] = 4.2, 95% CI 2.3–7.6%; *P* = 0.0001), while the younger adolescents (aged 10–14 year) were more infected with *P. malariae* with a prevalence of 9%.

**Fig. 4:**
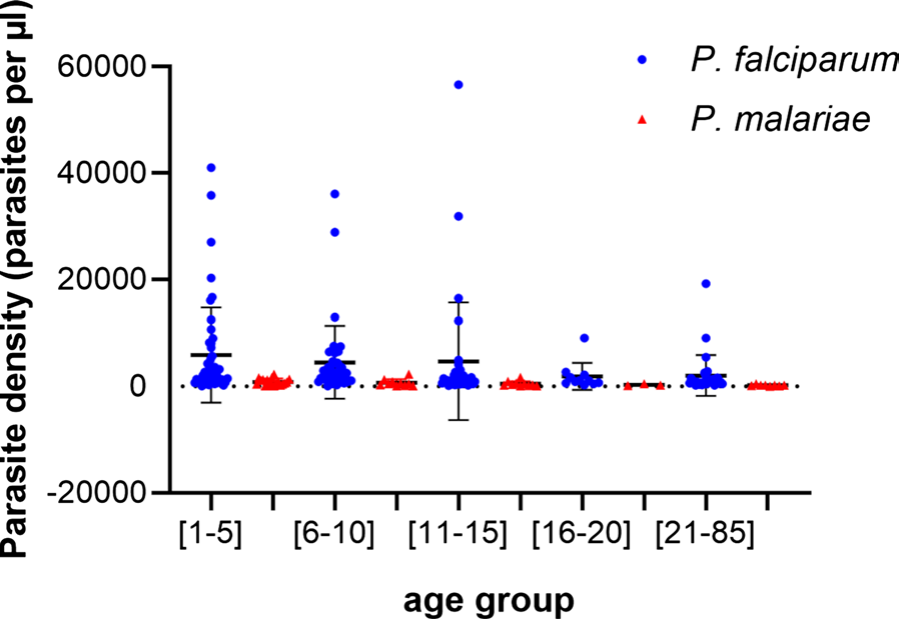
Parasite density by age groups

The overall geometric mean *P. falciparum* and *P. malariae* parasitaemia was 4354.7 ± 7991.6 and 606.1 ± 572.9 parasites/μl, respectively. The highest parasitaemia was recorded in children aged 1–5 years, at 5873.9 ± 8910.2 and 854.4 ± 608.4 parasites/μl for *P. falciparum* and *P. malariae,*, respectively. The lowest parasitaemia was observed in adults (age group: 21–85 years), at 1901.8 ± 2389 and 215 ± 119.8 parasites/μl for *P. falciparum* and *P. malariae*, respectively (Fig. 4). The mean of asexual parasite densities did not follow the that of prevalence of infection; hence, the density of *Plasmodium* infection increased significantly in children aged < 5 years compared to the other age groups (Fig. 4), decreasing from the younger age group to the older ones, leading to a significant reduction in the trophozoite density in subjects in the older age groups (Kruskal Wallis test,* H* = 20.03, *P* = 10^−3^).

## Discussion

In this study we assessed the transmission dynamics of *P. falciparum* and *P. malariae* infections in mosquito and human populations. The study produced strong evidence that malaria remains highly prevalent in the study locations in Cameroon.

### *An. funestus* is the predominant malaria vector in Central Cameroon

Three anopheline species were identified in the study area: *An. funestus* s.s., *An. gambiae* s.l. and *An. hancocki*, of which *An. funestus* s.s.(97.3%) was the most prevalent species. Our results are similar to those obtained by Tchouakui et al. in Mibellon [29], where there was 100% *An. funestus* prevalence. Our findings vary slightly from those of Tchouakui et al. [21] who recorded 95% *An. funestus* s.s. in the same village and from those of Menze et al. [30] who observed 80.1% prevalence, possibly due to the season in which the mosquitoes were collected.

The focus on the collection of indoor blood-fed mosquitoes may have prevented us from collecting the outdoor resting species of subfamily Anophelinae. Otherwise, our results showed that *An. funestus* is the dominant species in this locality and that the prevalence could vary according on the season since the different collections did not take place during the same season. To the contrary, in Tibati, we recorded 94.4% *An. funestus* prevalence compared to 55.2 %and 16% prevalence in collections from 2015 and 2017, respectively, by Feufack-Donfack et al. [31]. This difference in mosquito distribution in Tibati could be due to the month of mosquito collection, as our work was conducted during the dry season when the temporary breeding sites necessary for *An. gambiae* development become scarce.

### High *Plasmodium* sporozoite and oocyst infection rates

Both *An. funestus* and *An. gambiae* were infected with *Plasmodium* spp. A slight increase in the overall infection rate was observed in *An. funestus* in the present study compared to samples collected in 2017 in Mibellon by Menze et al*.* [30] (5% vs 13.6%; *X*^2^ = 3.43, *P* = 0.06) and also to samples collected in Tibati in 2017 by Feufack-Donfack et al. [31] (5.8% vs 2.04%; *X*^2^ = 3.14, *P* = 0.07). Although not significant, this increase in sporozoite infection rate could be due to the reduction in efficacy of LLINs (approx. 3 years) that were massively distributed in this part of the country in 2015. The prevalence of infection was also low in another location.

The significantly higher overall infection rate found in the abdomen of the mosquitoes compared to the head + thorax (34.2% vs 12.3%; *X*^2^ = 48.98, *P* = 0.0001) and, in particular, the infection rate by *P. malariae* (8.2% vs 1.9%; *X*^2^ = 15.08, *P* = 0.0001) of mosquitoes collected in 2019 strongly support the role of physical barriers played by the mosquito midgut and immune genes in the process of oocyst migration to the salivary gland [32]. In addition, the large difference in sporozoite infection between *P. falciparum* and *P. malariae* could also be due to the lifespan of mosquitoes, which is generally no longer than 3 weeks in nature. In fact, *P. malariae* has a longer sporogonic cycle (21 days) [33] than *P. falciparum* (14 days), which may explain the reduced likelihood of finding *P. malariae* sporozoites in the mosquitoes. The study site of Mibellon had a high infection rate with *P. falciparum* and *P. malariae*; this can possibly be explained by the perennial presence of the malaria vector, mainly *An. funestus*, during the year in Mibellon. The high infection rate of oocysts and sporozoites was recorded among the mosquito population of Mibellon village despite the human factors, vector immunity, midgut microbiota [34] and midgut epithelium [35], all of which are the causes of the reduction in the number of malaria parasites in the mosquito host. These results point to Mibellon as the area of high malaria endemicity and suggest that this location and surrounding villages are suitable for parasitological investigation of *P. malariae* in the human population.

### Multispecies malaria infection prevalence in human population

The high prevalence of infection and high parasitaemia indicate that the Ngatti health district is a high-risk zone for malaria transmission. This is supported by earlier reports of the prevalence of malaria infection in the Adamawa Region varying from 8.1% to 10.6% in symptomatic children aged between 2 and 9 years [5, 20, 31]. Moreover, the mode of malaria transmission is known to be influenced by environmental factors, such as temperature, rainfall and humidity. The high sporozoite infection rate is the main contributor to the maintenance of malaria endemicity in Mibellon. The 64.7% prevalence of malaria infection recorded in this study was lower than expected. Abdulraheem et al. [36] recorded a high prevalence (80%) of malaria in the asymptomatic population in Nigeria using PCR; this prevalence was almost twofold higher than that obtained by microscopy (47%). Similar results were obtained in the DRC by Nundu et al. [15], who recorded 62% and 33% infection rate of asymptomatic individuals using PCR and microscopy, respectively. The low *Plasmodium* spp. infection rate recorded in the present study could be due to several factors, such as the sampling season and the molecular method used for species identification. The PCR test used in this study targeted the 18S rRNA gene [28] whereas the studies cited above targeted the mt gene* cox* III [37] in the PCR assays. Unlike the standard 18S rRNA PCR method, the* cox* III method is able to detect an additional 10–50% more cases with high sensitivities for all four *Plasmodium* species due to the high number of parasite copies (20–150) found in* cox* III compared to 18S (4–8 copies) [37].

The results also revealed an association between the prevalence of malaria and age at infection. The highest prevalence of malaria (*P. falciparum* and Plasmodium mixed) infection was recorded in children in the age group 6–10 years. Regarding this result, it should be noted that malaria control campaigns target children aged < 5 years of age and pregnant women, with parents advised to ensure that children aged < 5 years, who are considered at high risk of contracting malaria, sleep under a LLIN. This possibly explains the reduction in the prevalence of infection among children in this young age group. However, the good use of ITNs for children aged < 5 years may impede or delay the development of malaria protective immunity in children aged 6–10 years [38, 39]. Also, the prevalence of infection with *P. falciparum* decreased significantly with age from 10 to 85 years, which could be accounted for by the acquisition of protective immunity by adolescents and adults following multiple exposures to malaria infection [40].

An estimation of malaria parasite density is necessary for patient management. Our study revealed that asymptomatic people coming from different villages in the Ngatti health district tolerated the high burden of asexual parasitaemia of up to 56,600 parasites/μl blood for *P. falciparum* without manifesting any sign of the disease; this burden could be more if we combine the parasitaemia in the case of co-infection. *Plasmodium falciparum* and *P. malariae* parasitaemia were significantly associated with age (*P* < 0.001) and, not surprisingly, the highest parasitaemia was recorded in children aged < 5 years. This result is explained by the low levels of immunity in young children and increasing naturally acquired immunity in older children through repeated exposure to the parasite [41, 42].

The prevalence of *P. malariae* obtained by microscopy was low, although higher than the general prevalence observed in Cameroon (3%) [5]. The highest prevalence was also observed in individuals between 11 and 20 years old. According to the “niche theory”, the elimination of *P. falciparum* in the lower age groups can be assumed to allow non-falciparum species, such as *P. malariae*, to become more important as *P. falciparum* is eliminated. In general, high transmission areas constitute a significant infectious reservoir of malaria parasites due to the potentially high percentage of asymptomatic malaria parasite carriers in this location [13, 14], which is what was observed in the Ngatti district health.

## Conclusion

The aim of this study was to examine the prevalence of malaria infection, targeting both *P. falciparum* as well as *P. malariae*, in the asymptomatic population. The findings revealed that malaria endemicity in the Adamawa Region of Cameroon is driven by multiple *Plasmodium* species, although *P. falciparum* remains the most prevalent. In addition, *P. malariae* infection and *P. falciparum/P. malariae* co-infections are both transmitted to humans by *An. funestus* in the studied localities and play an important role in malaria transmission. We found that older children were the age group most infected with malaria. Thus, beyond the control of malaria targeting only the under-fives, pregnant women and *P. falciparum*, a comprehensive policy targeting all age groups of the population should be put in place to eliminate malaria and its reservoir species more effectively.

## Data Availability

All data are fully available without restriction.

## Abbreviations

ACT: Artemisinin-based combination therapy
IRS: Indoor residual spraying
ITNs: Insecticide treated nets
LLINs: Long-lasting insecticide-treated bed nets
*P. OVM*: *Plasmodium malariae*,* Plasmodium ovale* OR *Plasmodium vivax*
RDTs: Rapid diagnostic tests
